# Artificial Intelligence-Based, Wavelet-Aided Prediction of Long-Term Outdoor Performance of Perovskite Solar Cells

**DOI:** 10.1021/acsenergylett.4c00328

**Published:** 2024-03-19

**Authors:** Ioannis Kouroudis, Kenedy Tabah Tanko, Masoud Karimipour, Aziz Ben Ali, D. Kishore Kumar, Vediappan Sudhakar, Ritesh Kant Gupta, Iris Visoly-Fisher, Monica Lira-Cantu, Alessio Gagliardi

**Affiliations:** † Department of Electrical Engineering, School of Computation, Information and Technology, Technical University of Munich, Hans-Piloty Strasse 1, 85748 Garching bei Munich,Germany; ‡ Catalan Institute of Nanoscience and Nanotechnology (ICN2), CSIC and The Barcelona Institute of Science and Technology, 08193 Bellaterra, Barcelona, Spain; ¶ Ben-Gurion Solar Energy Center, Swiss Inst. for Dryland Environmental and Energy Research, The Jacob Blaustein Institutes for Desert Research (BIDR), Ben-Gurion University of the Negev, Sede Boker Campus, Midereshet Ben-Gurion 84990, Israel; § Munich Data Science Institute, TUM, 85748 Garching, Walther-von-Dyck-Straße 10, Germany

## Abstract

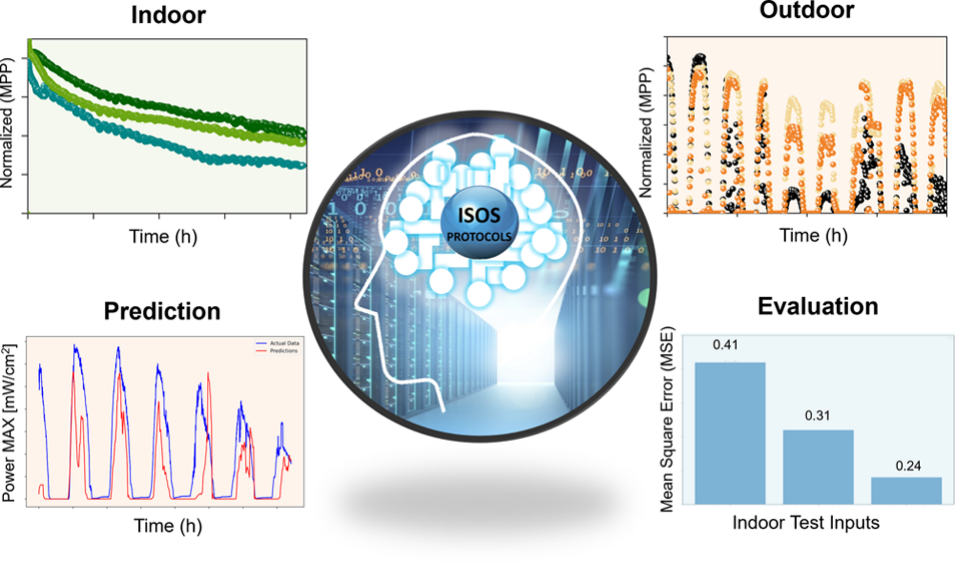

The commercial development of perovskite solar cells (PSCs) has been significantly delayed by the constraint of performing time-consuming degradation studies under real outdoor conditions. These are necessary steps to determine the device lifetime, an area where PSCs traditionally suffer. In this work, we demonstrate that the outdoor degradation behavior of PSCs can be predicted by employing accelerated indoor stability analyses. The prediction was possible using a swift and accurate pipeline of machine learning algorithms and mathematical decompositions. By training the algorithms with different indoor stability data sets, we can determine the most relevant stress factors, thereby shedding light on the outdoor degradation pathways. Our methodology is not specific to PSCs and can be extended to other PV technologies where degradation and its mechanisms are crucial elements of their widespread adoption.

Single-junction perovskite-based solar cells (PSCs) have demonstrated certified power conversion efficiencies (PCEs) above 26%.^1^ With PCEs on par with those of well-established commercial PV technologies, the research focus is now aimed at improving PSCs’ operational stability, which is approximately 20 years for commercial silicon PV. Although a consensus on accelerated stability tests including various stress factors has been published,^2^ a strategy that permits the prediction of the PSCs’ outdoor performance and lifetime from accelerated indoor aging tests is currently missing. Operational conditions outdoors include diurnal light/dark cycling and varying temperatures, illumination intensity, and spectrum during sunlight hours. While the PSC performance dependence on each of these factors was determined, it is highly sensitive to combinations of them,^3^ making predictions of outdoor daily energy yield rather complex.^4^ Further complication arises from well-known PSC transient performance variations on time scales from seconds to days, superimposed on nonreversible, long-term degradation.^5−7^

Several attempts to predict PSC lifetimes based on indoor tests have been published, such as testing under constant illumination,^8^ a combination of continued 1 sun illumination (ISOS-L1 protocol) tests and dark storage (ISOS-D protocol) tests,^9^ or damp heat testing at 85 °C and 85% relative humidity.^10^ These works predicted lifetimes of 5–20 years for PSCs of various architectures, demonstrating the need for accelerated testing. A recent publication described good correlation between modeling based on PSC indoor tests of light intensity and temperature-dependent performance dynamics and its long-term, nonreversible outdoor degradation.^11^ Several studies simulated outdoor conditions in lab tests.^12,13^ However, direct predictions of the detailed time- and climate conditions- dependent outdoor PSC performance including both daily power output and nonreversible degradation, based on accelerated indoor tests, are currently lacking.

Machine learning (ML) is a specialized area within artificial intelligence that focuses on the development of algorithms that acquire knowledge and make predictions or decisions by discerning patterns and insights within the data used for their training. ML can be applied to the development of solar cells, including PSCs, in predicting material composition–properties relations, optimization of device structure and fabrication processes, and reconstruction of measurement data.^14−23^ Few published works utilized ML tools to study factors affecting PSC stability.^24,25,25^ Herein we apply multiple ML algorithms for correlating indoor and outdoor performance testing of PSCs, successfully predicting outdoor time- and weather-dependent PCE patterns based on indoor constant illumination (ISOS-L) tests. The crucial aspect of the pipeline lies in training the algorithm with a different combination of indoor tests. Thus, we provide a robust way of determining the relevant outdoor degradation factors from specialized indoor accelerated tests.

The experimental data are generated from devices similarly fabricated and aged indoors and outdoors in two different laboratories: BGU and ICN2. PSCs were fabricated in the *nip* configuration of FTO/c-TiO_2_/m-TiO_2_/CsMAFAPb(IBr)3/Spiro-OMeTAD/Au, and the solar cells were subjected to indoor (constant illumination, in Air or N_2_) and outdoor photostability tests at maximum power point (MPP, encapsulated) conditions using an MPP tracker. The details of device fabrication and testing are provided in the Supporting Information. Photographs of the devices are shown in Figure S1. Further, herein we present the results of the best performing machine learning algorithms. The results of additional algorithms can be found in the Supporting Information.

The core of our method is the prediction of the outdoor behavior given the outdoor environmental conditions and a set of indoor accelerated degradation tests, at different light intensities and environments (Figure 1a). Each indoor test contains a level of relevance to the outdoor behavior, as well as a level of overlap with other indoor tests. This is not always trivial to determine, which hinders the analysis of real-world degradation through accelerated tests. Our pipeline attempts to account for both effects. Initially, the prediction algorithm was trained using a single type of indoor test as input. This was repeated for all indoor tests. The test that produced the lowest error, i.e., the difference between the predicted and actual outdoor performance tracks, bears the most relevance to the outdoor degradation mechanisms. As a second step the algorithm was trained using combinations of indoor tests as input. Training a machine learning algorithm with data that are strongly correlated leads to either inefficiency or at worse an accuracy decrease in the prediction. However, viable combinations of uncorrelated inputs can significantly enhance the quality of the predictions. To this end, we have implemented a Frechet distance metric^26^ that determines whether two curves are correlated or not. This is achieved taking into account both the positions and the ordering of the curve points to account for stretching effects. The prediction errors and their relation to the errors of the previous step allowed us to determine dependencies between indoor tests. The entire pipeline is summarized in Figure 1a. This pipeline has two major outcomes. The first is a robust prediction of outdoor behavior based on indoor tests, combined with the actual environmental conditions of the area the panels were deployed. The second is the determination of the most relevant indoor tests for outdoor behavior prediction and their interdependence.

**Figure 1:**
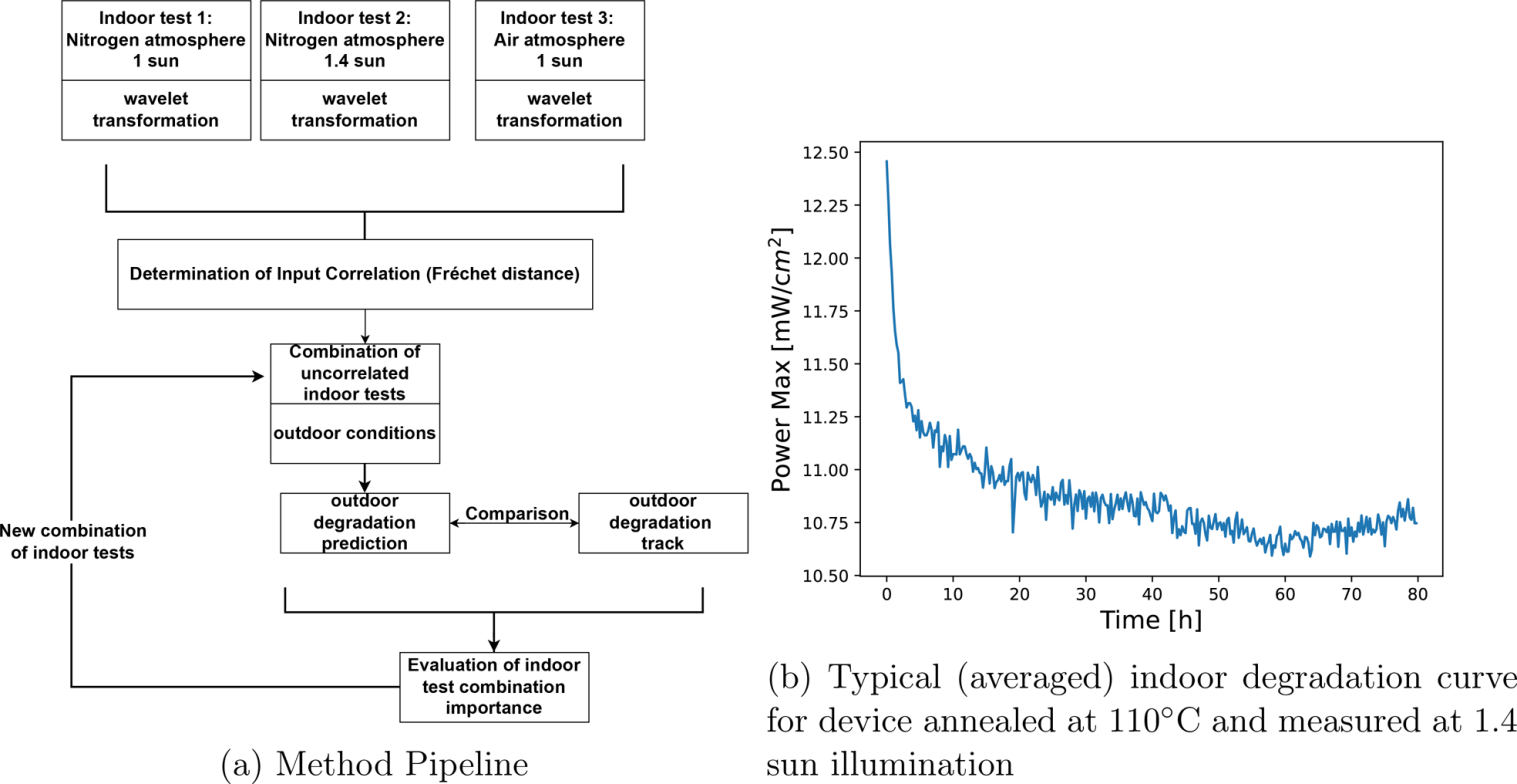
Methodology (a) and typical input (b).

The measurements of maximum power output versus time (see Figure 1b for a representative curve) were performed on devices fabricated with six different annealing temperatures. Since annealing temperature has been shown to affect the final PCE and by extension the maximum power output,^27^ altering this value provides a natural data set that allows for different behaviors. Four identical devices were fabricated per annealing temperature to ensure the reproducibility of results. The performance tracks (maximum power output vs time) of PSCs devices with the same fabrication process were averaged to decrease the noise to signal ratio as much as possible. This process was applied both to the outdoor and indoor curves. This allowed us to capture the average trend but entirely discards the intrinsic deviations that are present in the perovskite fabrication process. To mitigate this, a follow-up work will include uncertainty quantification of predictions as well as the predictions themselves. Six unique data points present a challenging start for any machine learning endeavor, while using all 24 would involve highly noisy measurements. To ensure that the algorithm was fairly tested despite the limited numbers of samples, we implemented a 6-fold validation strategy as outlined in Figure S2. Additionally, during the measurements of the outdoor conditions (irradiance and temperature), the sensor failed for some hours. To fill the data gaps, a K-nearest neighbor data imputation method was employed, along with a classification that determined the night and day cycle and set the irradiance values to zero during the night. Lastly, to extract more concentrated information from the data, we transformed the time series using the Daubechies 2 wavelet. The wavelet transformation was favored over the Fourier one as the time series in question show a degrading character which would not be present at the pure frequency domain. Short-time Fourier transform was also considered over wavelets, but the wavelet’s flexibility of representation ultimately proved a crucial advantage that enhanced predictions significantly. Multiple wavelets were tested, and the final choice of the specific wavelet was done based on the k-fold test set prediction error.

At present, the best performing method was proven to be Kernel Ridge Regression (KRR).^28^ It combines the kernel trick and L2 regularization with a linear regression algorithm. The kernel trick is the process of projecting the data into a more informative data space by means of a kernel function. An additional term is added to the error expression, specifically, the euclidean norm of the model parameters, to counter overfitting. The parameters of the models were optimized using Bayesian optimization, which outperformed the next best hyperparameter tuning method by at least 20%. This optimization provides a very efficient way of determining the hyperparameter values as it learns from previous hyperparameters and makes a more educated guess after every iteration. This ensures both faster convergence and higher probability at finding a better optimum point. The loss function chosen was the Mean Square Error. This can be interpreted as the average squared distance of every true point from its predicted relevant value. The methods have been implemented in Python with the sklearn library.^29^ Multiple additional algorithms were tested, notably Gaussian Processes,^30^ bidirectional Long Short-term Memory Networks,^31^ and Transformers.^32^ The results can be seen in the SI in Figures S3–S5. After the Frechet distance is calculated, the results can be seen in Table 1. As a cut-off we have chosen 0.95 and will therefore not test the combinations of 1.4 sun and 1 sun as they are considered highly correlated and therefore redundant.

**Table 1:** Frechet Distance Denoting Curve Similarity^a^

	1.4 sun in N_2_	1 sun in N_2_	1.0 sun in air
1.4 sun in N_2_	1	0.96	0.86
1 sun in N_2_	0.96	1	0.89
1 sun in air	0.86	0.89	1

a 1 indicates identical curves.

Since we expect a certain smoothness from the results, they have been denoised using Impulse Response (IIR) filter. The first half of the core results are shown in Figure 2, reflecting the relative relevance of the indoor tests in relation to the outdoor behavior. Specifically, encapsulated devices were tested outdoors, while nonencapsulated devices were tested in the indoor setup in air or N_2_ atm. Therefore, it is natural to expect that the indoor tests performed in air have the least relevance to the outdoor tests. This is indeed verified with the air measurements (indoor test 3 in Figure 1a) generating predictions that are more than twice worse than the best ones. Further, the light intensity of 1 sun is expected to be the most relevant indoor tests. This is consistent with the results presented, with the 1 sun in nitrogen (indoor test 1) results outperforming the next best by 30%. As is evident, our pipeline provides not only qualitative evaluations but also quantitative ones, which allows for the precise determination of the stress factor importance. Using the conformity of the results to our expectations as proof of concept, we can now expand the algorithm to tests that are nontrivially correlated with the outdoor behavior in a future work. Compounding on this, if tests of combined different stress factors provide better accuracy than separate ones, then we can assume that the degradation paths are nontrivially intertwined, which will affect our rationalization of the mechanism.

**Figure 2:**
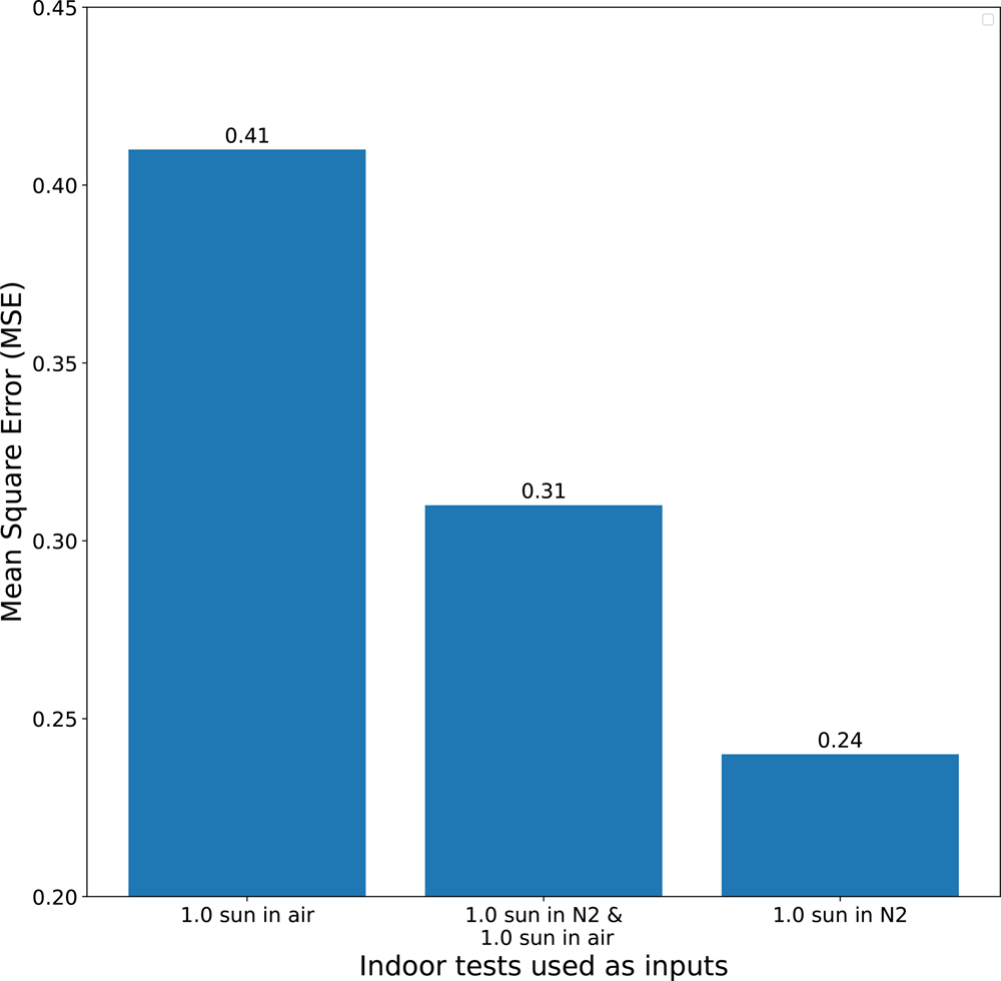
Comparative accuracy of predictions with different indoor tests as inputs.

The second half of the core results are shown in Figure 3a and provide a strong proof that the algorithm has learned the real device behavior. The algorithm reconstruction of the outdoor behavior is quite remarkably accurate with an average mean square error of 0.24, and the reconstructed curve fits almost perfectly the measured one, in data that is not used during training. Further, as can be seen in Figure 3b, when the trained algorithm was presented with test data generated from another lab, without UV protection during aging (see the Supporting Information) and with significantly different environmental conditions (Mediterranean and desert climates at ICN2 and BGU, respectively), the prediction was accurate within 1 order of magnitude. In fact, the discrepancy could be credibly attributed to the differences in aging protocols, especially the lack of UV protection. This is despite the fact that the indoor measurements at ICN2 were conducted without UV light. The existence of double peaks in the prediction curve of Figure 3b can be attributed to the existence of a high-frequency noise component from the wavelet decomposition due to the difference in measuring frequencies across laboratories. This part of the pipeline not only verifies the results of the first part shown in Figure 2 but also provides important value in the form of predicting outdoor PCE evolution. It further proves that so long as the indoor and outdoor measurements are consistent with each other, a generally well-behaving prediction can be expected. By utilizing this functionality, the stability of new device types can be evaluated in a matter of days rather than months.

**Figure 3:**
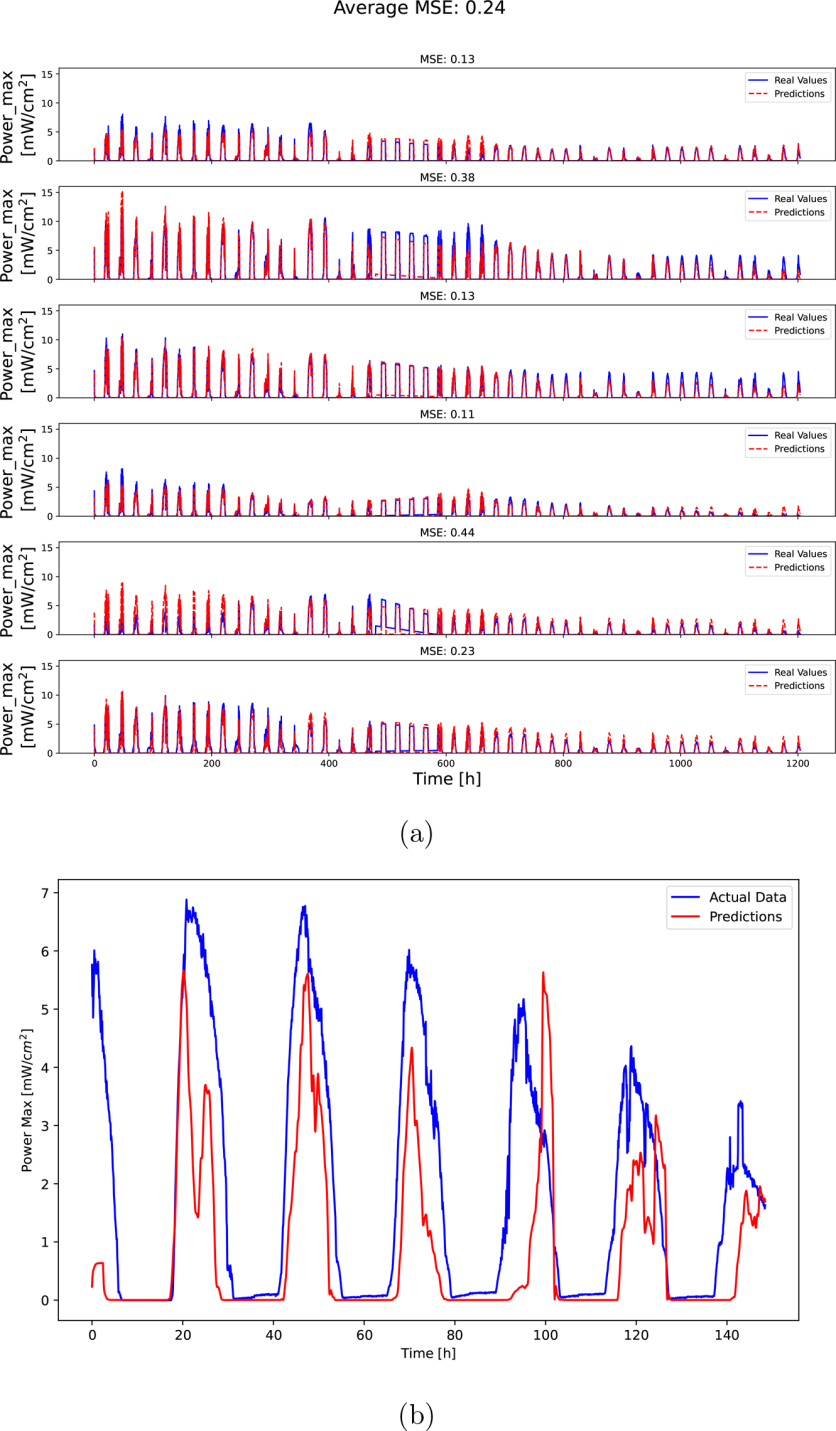
Predictions (red) and true (blue) performance tracks of maximum power evolution in outdoor conditions. The true tracks are generated by averaging over the measured curves of all the devices with the same fabrication procedure. (a) Tracks measured in Barcelona, Spain, and (b) tracks measured in Sede Boqer, Israel

In conclusion, we have presented a robust pipeline that identifies the relevant stress factors of perovskite solar cell degradation in a qualitative and quantitative fashion. Further, it allows direct predictions of outdoor solar cell stability based on accelerated indoor stability tests without further modeling. Subsequently, the same pipeline is used to reconstruct the outdoor behavior based only on the relevant indoor tests. These findings are extremely important, as they can rationalize outdoor degradation mechanisms from relatively quick tests as well as provide insight into the degradation mechanisms as a whole. Further, given the quantitative nature of our factor importance, the laboratories can choose to test a device with a less relevant indoor test if they judge that the accuracy loss is sufficiently compensated by the decreased test duration. Further, by identifying the relevant stress factors, the syntheses can move toward the direction that mitigates these specific effects. The pipeline’s results were achieved using only six different device types to train the algorithms. Nevertheless, with an expanded data set, we could have efficiently utilized more complex algorithms such as Transformers and bidirectional Long Short-Term Memory (bLSTM) networks. These algorithms have proven robust and accurate when trained with large data sets. In a data set of the size that we are currently investigating, the additional complexity is proven to decrease the quality of the prediction. In contrast, the kernel methods have a lower parametric load and are therefore better suited to our case study. Our pipeline can be applied without loss of generality both to low-throughput and high-throughput laboratories by choosing a suitable method. Since our pipeline is general and can be applied across technologies and laboratories, it can be used to provide general intuition that will combine the findings of many independent and disjoint laboratories.

## Supporting Information Available

Experimental Methods (preparation of triple cation perovskite precursor solution, perovskite solar cell fabrication, encapsulation, and indoor and outdoor photostability studies) and Algorithms and Data Processing Methods (algorithm testing strategy and results of different algorithms) (PDF)

## References

[ref1] National Renewable Energy Laboratory. Best research-cell efficiencies chart https://www.nrel.gov/pv/assets/pdfs/best-research-cell-efficiencies.pdf (accessed Feb 29, 2024).

[ref2] Khenkin M. V.; Katz E. A.; Abate A.; Bardizza G.; Berry J. J.; Brabec C.; Brunetti F.; Bulović V.; Burlingame Q.; Di Carlo A.; others; et al. Consensus statement for stability assessment and reporting for perovskite photovoltaics based on ISOS procedures. Nature Energy 2020, 5, 35–49. 10.1038/s41560-019-0529-5.

[ref3] Ali M. U.; Mo H.; Li Y.; Djurišić A. B. Outdoor stability testing of perovskite solar cells: Necessary step toward real-life applications. APL Energy 2023, 1, 020903 10.1063/5.0155845.

[ref4] Jošt M.; Lipovšek B.; Glažar B.; Al-Ashouri A.; Brecl K.; Matič G.; Magomedov A.; Getautis V.; Topič M.; Albrecht S. Perovskite solar cells go outdoors: field testing and temperature effects on energy yield. Adv. Energy Mater. 2020, 10, 2000454 10.1002/aenm.202000454.

[ref5] Domanski K.; Alharbi E. A.; Hagfeldt A.; Grätzel M.; Tress W. Systematic investigation of the impact of operation conditions on the degradation behaviour of perovskite solar cells. Nature Energy 2018, 3, 61–67. 10.1038/s41560-017-0060-5.

[ref6] Khenkin M. V.; KM A.; Visoly-Fisher I.; Kolusheva S.; Galagan Y.; Di Giacomo F.; Vukovic O.; Patil B. R.; Sherafatipour G.; Turkovic V.; et al. others Dynamics of photoinduced degradation of perovskite photovoltaics: from reversible to irreversible processes. ACS Applied Energy Materials 2018, 1, 799–806. 10.1021/acsaem.7b00256.

[ref7] Tress W.; Yavari M.; Domanski K.; Yadav P.; Niesen B.; Baena J. P. C.; Hagfeldt A.; Graetzel M. Interpretation and evolution of open-circuit voltage, recombination, ideality factor and subgap defect states during reversible light-soaking and irreversible degradation of perovskite solar cells. Energy Environ. Sci. 2018, 11, 151–165. 10.1039/C7EE02415K.

[ref8] Zhao X.; Liu T.; Burlingame Q. C.; Liu T.; Holley R. III; Cheng G.; Yao N.; Gao F.; Loo Y.-L. Accelerated aging of all-inorganic, interface-stabilized perovskite solar cells. Science 2022, 377, 307–310. 10.1126/science.abn5679. 35709247

[ref9] Liu T.; Zhao X.; Wang P.; Burlingame Q. C.; Hu J.; Roh K.; Xu Z.; Rand B. P.; Chen M.; Loo Y.-L. Highly transparent, scalable, and stable perovskite solar cells with minimal aesthetic compromise. Adv. Energy Mater. 2023, 13, 2200402 10.1002/aenm.202200402.

[ref10] Kobayashi E.; Tsuji R.; Martineau D.; Hinsch A.; Ito S. Light-induced performance increase of carbon-based perovskite solar module for 20-year stability. Cell Reports Physical Science 2021, 2, 100648 10.1016/j.xcrp.2021.100648.

[ref11] Jiang Q.; Tirawat R.; Kerner R. A.; Gaulding E. A.; Xian Y.; Wang X.; Newkirk J. M.; Yan Y.; Berry J. J.; Zhu K. Towards linking lab and field lifetimes of perovskite solar cells. Nature 2023, 623, 313 10.1038/s41586-023-06610-7. 37696288

[ref12] Song W.; Aernouts T. Novel test scenarios needed to validate outdoor stability of perovskite solar cells. Journal of Physics: Energy 2020, 2, 021003 10.1088/2515-7655/ab6008.

[ref13] Tress W.; Domanski K.; Carlsen B.; Agarwalla A.; Alharbi E. A.; Graetzel M.; Hagfeldt A. Performance of perovskite solar cells under simulated temperature-illumination real-world operating conditions. Nature energy 2019, 4, 568–574. 10.1038/s41560-019-0400-8.

[ref14] Li F.; Peng X.; Wang Z.; Zhou Y.; Wu Y.; Jiang M.; Xu M. Machine learning (ML)-assisted design and fabrication for solar cells. Energy & Environmental Materials 2019, 2, 280–291. 10.1002/eem2.12049.

[ref15] Srivastava M.; Howard J. M.; Gong T.; Rebello Sousa Dias M.; Leite M. S. Machine learning roadmap for perovskite photovoltaics. J. Phys. Chem. Lett. 2021, 12, 7866–7877. 10.1021/acs.jpclett.1c01961. 34382813

[ref16] Yılmaz B.; Yıldırım R. Critical review of machine learning applications in perovskite solar research. Nano Energy 2021, 80, 105546 10.1016/j.nanoen.2020.105546.

[ref17] Tao Q.; Xu P.; Li M.; Lu W. Machine learning for perovskite materials design and discovery. npj Computational Materials 2021, 7, 23 10.1038/s41524-021-00495-8.

[ref18] Liu Z.; Rolston N.; Flick A. C.; Colburn T. W.; Ren Z.; Dauskardt R. H.; Buonassisi T. Machine learning with knowledge constraints for process optimization of open-air perovskite solar cell manufacturing. Joule 2022, 6, 834–849. 10.1016/j.joule.2022.03.003.

[ref19] Sun S.; Hartono N. T.; Ren Z. D.; Oviedo F.; Buscemi A. M.; Layurova M.; Chen D. X.; Ogunfunmi T.; Thapa J.; Ramasamy S.; et al. Accelerated development of perovskite-inspired materials via high-throughput synthesis and machine-learning diagnosis. Joule 2019, 3, 1437–1451. 10.1016/j.joule.2019.05.014.

[ref20] Taherimakhsousi N.; Fievez M.; MacLeod B. P.; Booker E. P.; Fayard E.; Matheron M.; Manceau M.; Cros S.; Berson S.; Berlinguette C. P. A machine vision tool for facilitating the optimization of large-area perovskite photovoltaics. npj Computational Materials 2021, 7, 190 10.1038/s41524-021-00657-8.

[ref21] Harth M.; Vesce L.; Kouroudis I.; Stefanelli M.; Di Carlo A.; Gagliardi A. Optoelectronic perovskite film characterization via machine vision. Sol. Energy 2023, 262, 111840 10.1016/j.solener.2023.111840.

[ref22] Lampe C.; Kouroudis I.; Harth M.; Martin S.; Gagliardi A.; Urban A. S. Rapid Data-Efficient Optimization of Perovskite Nanocrystal Syntheses through Machine Learning Algorithm Fusion. Adv. Mater. 2023, 35, 2208772 10.1002/adma.202208772. 36681859

[ref23] Mayr F.; Harth M.; Kouroudis I.; Rinderle M.; Gagliardi A. Machine Learning and Optoelectronic Materials Discovery: A Growing Synergy. J. Phys. Chem. Lett. 2022, 13, 1940–1951. 10.1021/acs.jpclett.1c04223. 35188778

[ref24] Odabaşı Ç.; Yıldırım R. Machine learning analysis on stability of perovskite solar cells. Sol. Energy Mater. Sol. Cells 2020, 205, 110284 10.1016/j.solmat.2019.110284.

[ref25] Graniero P.; Khenkin M.; Köbler H.; Hartono N. T. P.; Schlatmann R.; Abate A.; Unger E.; Jacobsson T. J.; Ulbrich C. The challenge of studying perovskite solar cells’ stability with machine learning. Frontiers in Energy Research 2023, 11, 1118654 10.3389/fenrg.2023.1118654.

[ref26] Har-Peled S.; Raichel B. The Fréchet distance revisited and extended. ACM Transactions on Algorithms (TALG) 2014, 10, 1–22. 10.1145/2532646.

[ref27] Lin L.; Ravindra N. Temperature dependence of CIGS and perovskite solar cell performance: an overview. SN Applied Sciences 2020, 2, 1361 10.1007/s42452-020-3169-2.

[ref28] Zhang Y.; Duchi J.; Wainwright M. Divide and conquer kernel ridge regression. Proc. Conference on learning theory 2013, 592–617.

[ref29] Pedregosa F.; et al. Scikit-learn: Machine Learning in Python. Journal of Machine Learning Research 2011, 12, 2825–2830.

[ref30] Williams C. K.; Rasmussen C. E. Gaussian processes for machine learning; MIT Press: Cambridge, MA, 2006; Vol. 2.

[ref31] Huang Z.; Xu W.; Yu K. Bidirectional LSTM-CRF models for sequence tagging (accessed 2024-2-29). arXiv 2015, 1508.01991 10.48550/arXiv.1508.01991.

[ref32] Vaswani A.; Shazeer N.; Parmar N.; Uszkoreit J.; Jones L.; Gomez A. N.; Kaiser L. u.; Polosukhin I. Attention is All you Need. Advances in Neural Information Processing Systems, https://proceedings.neurips.cc/paper_files/paper/2017/file/3f5ee243547dee91fbd053c1c4a845aa-Paper.pdf, (accessed 2024-2-29) 2017.

