# Supplementary material for: Artificial Intelligence-Based, Wavelet-Aided Prediction of Long-Term Outdoor Performance of Perovskite Solar Cells
Source: ACS Energy Lett. 2024 Mar 19;9(4):1581–6. doi: 10.1021/acsenergylett.4c00328 (PMC11019640; doi:10.1021/acsenergylett.4c00328)
Supplement: Supplementary file 1 — nz4c00328_si_001.pdf [file nz4c00328_si_001.pdf]

Artificial intelligence-based, wavelet aided prediction of long-term outdoor performance of perovskite solar cells

Ioannis Kouroudis, ∗,†,‖ Kenedy Tabah Tanko,‡,‖Masoud Karimipour,‡ Aziz Ben Ali,† D. Kishore Kumar,¶ Vediappan Sudhakar,¶ Ritesh Kant Gupta,¶ Iris Visoly-Fisher,¶ Monica Lira-Cantu,‡ and Alessio Gagliardi†,§

†Department of Electrical and Computer Engineering,Technical University of Munich,85748 Garching bei Munich,Germany

‡Catalan Institute of Nanoscience and Nanotechnology (ICN2), CSIC and The Barcelona Institute of Science and Technology, Bellaterra, Barcelona, Spain

¶ Ben-Gurion Solar Energy Center, Swiss Inst. for Dryland Environmental and Energy Research , The Jacob Blaustein Institutes for Desert Research (BIDR), Ben-Gurion University of the Negev, Sede Boker Campus, Midereshet Ben-Gurion 84990, Israel

§Munich Data Science Institute, TUM, Garching, Walther-von-Dyck-Straße 10 (Germany)

‖this authors have contributed equally

E-mail: irisvf@bgu.ac.il; monica.lira@icn2.cat; alessio.gagliardi@tum.de

SUPPLEMENTARY INFORMATION

Experimental Methods

1. **Preparation of triple cation perovskite precursor solution**

At BGU: Precursors: FK209 Co(III) TFSI salt (>99%) was obtained from Lumtec, Spiro-OMeTAD (99%), Bis(trifluoromethane)sulfonimide lithium salt (LiTFSi, 99.95%), tert-butyl pyridine (tBP, 98%) were obtained from Merck. Formamidinium iodide (FAI, >99.99%), methylammonium bromide (MABr, >99.99%) were obtained from Greatcell, Cesium iodode (CsI, >99.0%), lead iodide (PbI2, 99.99%) and lead bromide (PbBr2, >98.0%) were obtained from TCI. These materials were used without further purification. Dry solvents (over molecular sieves): 99.8 % dimethylformamide was purchased from Acros Organics, 99.8 %

cholorobenzene, 99.9 % Acetonitrile and 99.7+ % dimethyl sulfoxide (DMSO) were purchased from Thermo Scientific.

A 1.5M solution of PbI2 was prepared by adding 691.5 mg of PbI2 to 1000 µL of 4:1 vol. ratio of DMF and DMSO. A 1.5 M solution of PbBr2 was prepared by adding 110.1 mg of PbBr2 to 200 µL of 4:1 vol. ratio of DMF and DMSO. Subsequently, the vials were heated to 180 ºC for 30 minutes. After cooling, 236.63 mg of FAI was added to the vial containing PbI2, and 30.81 mg of MABr was added to the vial containing PbBr2. These mixtures were stirred at 70 ºC for 10 minutes. Next, the formamidinium lead iodide (FAPbI3) solution was added to the vial containing methylammonium lead bromide (MAPbBr3). The resulting mixture was stirred at 70 ºC for 10 minutes. 5vol% of a 1.5M cesium iodide (CsI) solution in DMSO (60 µL), preheated to 150 ºC for 10 minutes, was added to the double cation perovskite precursor solution (1.2 mL). Finally, the triple cation perovskite precursor solution was stirred at 70 ºC for two hours before further usage. These procedures were performed in a N2-filled glovebox, following a previously published protocol.[1]

At ICN2: Materials. N, N-dimethylformamide (DMF, anhydrous, 99.8%), dimethyl sulfoxide (DMSO, anhydrous, 99.8%), chlorobenzene (anhydrous, 99.8%), acetonitrile (anhydrous, 99.8%), isopropanol (99.97%, anhydrous), and 4-tert-butylpyridine (TBP, 96%) were received from Merck Co. lead (II) bromide ($PbBr_2$, 99.99%), lead (II) iodide (PbI_2, 99.99%), formamidinium iodide (FAI), methylammonium iodide (MAI), and cesium iodide (CsI, 99.99%) were purchased from TCI chemicals. Spiro-OMeTAD (Spiro) was purchased from Xi'an Polymer Light Technology Corp. All chemicals are used as received without any further purification.

Firstly, PbI2/PbBr2 precursor solution was prepared by dissolving 548.6 mg of PbI_2, and 57.06 mg of $PbBr_2$ in 1mL of DMF:DMSO (4:1 volume ratio) at 150ºC for 5 min, then allowed to cool to room temperature. Thereafter, 27.02 mg of CsI, 178.94 mg of FAI, and 17.41 mg of MABr were weighed into a separate vial and dissolved in the PbI2/PbBr2 solution at 75ºC for 15 minutes to form the perovskite $Cs_{0.08}MA_{0.12}FA_{0.8}Pb(I_{0.88}Br_{0.12})_3$.

For the hole transport layer, 2,2',7,7'-Tetrakis[N,N-di(4-methoxyphenyl)amino]-9,9'-spirobifluorene (Spiro-OMeTAD, 60 mg, Aldrich) was dissolved in chlorobenzene (0.596 mL, CB). 4-tert-Butylpyridine (23µl, TBP) and tris(2-(1Hpyrazol-1-yl)-4-tert-butylpyridine) cobalt(III) tri[bis- (trifluoromethane)sulfonimide] (FK209, Aldrich) precursor solution (4.5 µl from 375 mg in 1 mL acetonitrile) and Lithium bis(13.5 µl, trifluoromethanesulfonyl) imide (Li-TFSI, Aldrich) precursor solution (100 mg in 378 mL acetonitrile) was added to the solution just before the deposition.

2. **Perovskite solar cell fabrication**

At BGU: Prior to the spin coating, fluorine-doped tin oxide (FTO) substrates (TEC 7, Pilkington, UK), were cleaned with a 2% Hellmanex-III solution, followed by deionized water, acetone, and isopropanol by ultrasonication. Subsequently, the FTO substrates were dried and exposed to UV-Ozone treatment for 30 minutes. The compact TiO2 layer on the FTO substrate was spin-coated using Ti-Nanoxide (Solaronix) at 5000 rpm for 30 seconds, followed by annealing in air at 550 ºC for 1 hour. The subsequent mesoporous TiO2 layer was prepared by mixing 150 mg (TiO2 Paste - DSL 30NR-D, Greatcell) in 1 mL of ethanol and stirring at room temperature overnight. After cooling, it was spin-coated at 4000 rpm for 10 seconds. The substrates were then annealed in a stepwise manner, at 125 °C for 5 minutes, 325 °C for 5 minutes, 375 °C for 5 minutes, and finally 450 °C for 30 minutes in air, followed by cooling to room temperature. 10 mg LiTFSi were dissolved in 1 mL of acetonitrile, spin-coated at 3000 rpm for 10 seconds and subjected to sequential annealing steps at 125 °C for 5 minutes, 325 °C for 5 minutes, 375 °C for 5 minutes, and 450 °C for 30 minutes. Finally, the substrates were treated with UV-Ozone for 5 minutes and immediately transferred into a nitrogen-filled glove-box.

All further steps were carried out in a nitrogen-filled glove-box. The perovskite precursor solution was spin-coated initially at 400 rpm for 10 seconds, followed by 4000 rpm for 30 seconds. The anti-solvent, chlorobenzene, was introduced by dripping it onto the substrate 10 seconds before the completion of the second spin-coating step. After spin-coating, the substrates were annealed at 100 °C for 60 min in the glovebox. Spiextracted_info_third_roundro-OMETAD solution was prepared by dissolving 122 mg of Spiro-OMETAD in 1 mL of chlorobenzene. 28.5 μL of tBP, 18 μL of LiTFSi, and 8 μL of FK209 Co(II)/Co (III) complex were added to this solution and thoroughly mixed. After cooling, the hole transport layer (HTL) was spin-coated at 4000 rpm for 20 seconds, by dripping onto the substrate 12 seconds before the completion of the spin-coating process. 80 nm thick gold electrode was deposited by thermal evaporation at a growth rate of 0.2-0.3 Å/s for the first 2 nm followed by 1.0-1.2 Å/s for the remaining 78 nm.

At ICN2: 24 FTO (16 Ω/cm2) substrates (2.5 cm×1.5 cm) were etched using Zinc powder and HCl 2M, then they were cleaned with 10% Hellmanex solution and then placed in bath sonicator with sequential cleaning using 2% Hellmanex solution, acetone, and isopropanol, for 30 min, 10 min, and 15 min, respectively. Then the substrates were exposed to UV/Ozone cleaner for 20 min and placed on hotplate, followed with heating up to 450 ºC for TiO_2 blocking layer deposition. TiO_2 precursor solution was prepared by mixing 9 mL ethanol (96%), 0.4 mL of acetyl acetone, and 0.6 mL of Titanuim diisopropoxide bis-acetyl acetonate, and it was sprayed directly on the pre-heated substrates during 6-8 minutes, then they were kept at 450 ºC for 30 min and thereafter were cooled down to room temperature. The mesoporous TiO_2 layer as electron transport layer (ETL) was also deposited using a solution containing 1mg of TiO_2 nanoparticles (30 nm) paste diluted in 6 g of ethanol. The solution was spin coated at 5000 rpm for 20 sec. and then substrates dried at 85 ºC and thereafter were annealed at 450 ºC for 30 min and cooled down naturally. At last, the substrates were exposed to UV-Ozone for 25 min and

transferred immediately to the controlled N_2 atmosphere glove box maintained at 18-20 ºC (H_2O: 1-1.5 ppm, O_2:1-3 ppm) for perovskite, interface modification and HTL layer deposition. 0.050 mL perovskite solution was spread over the substrates and span at 2000 rpm for 10 s and 6000 rpm for 30 s, and after 15 s of the second step, 0.01 mL ofextracted_info_third_round CB was injected instantly to the center of the substrate and after spinning, the film was quickly annealed. Here, six annealing temperatures were used: 100ºC (60 min), 110 ºC (50 min), 120 ºC (40 min), 130 ºC (25 min), 140 ºC (15 min), and 150 ºC (10 min), generating six device sets for stability testing. HTL layer was deposited by drop casting 45 µL of Spiro solution spinning at 3700 rpm for 20 sec. The 80 nm gold contact was deposited using a high vacuum PVD system with pressure below 8×10^{-7} atm.

3. **Encapsulation**

At BGU: Encapsulation was performed in ambient lab atmosphere. The perovskite and HTL layers were mechanically removed from the substrate edges. The contacts to the top and bottom electrodes were made with copper ribbons using silver paste and allowed to dry. The cells were shielded with Kapton tape so that the epoxy will not damage either perovskite or HTL. The epoxy and hardener (2,2 bis(4-(2,3epoxypropoxy)phenyl) propane (Nalmat-Trzebinia) were mixed at the ratio of 1:1 and applied to cover the entire cell. A glass slide of thickness 1 mm (Isolab, Germany) was then placed on the top and allowed to dry for 24h in dark. After curing, the metal pins were soldered to the copper ribbons as shown in the figure below.

At ICN2: The devices were encapsulated using our previously reported glass to glass encapsulation procedure.[2] Briefly, the active area of the solar cell was covered with a UV-curing epoxy and then the top glass was placed and exposed to a UV light source (254 nm) for 45 min and then copper tape was connected to the gold contacts without any other additive or epoxy and was used for the stability study. For the outdoor performance of the PSCs, 3-4 devices for each category were encapsulated and used the ISOS-O protocol study. The collected data was analyzed through obtained averaged values and standard errors for more clarity.

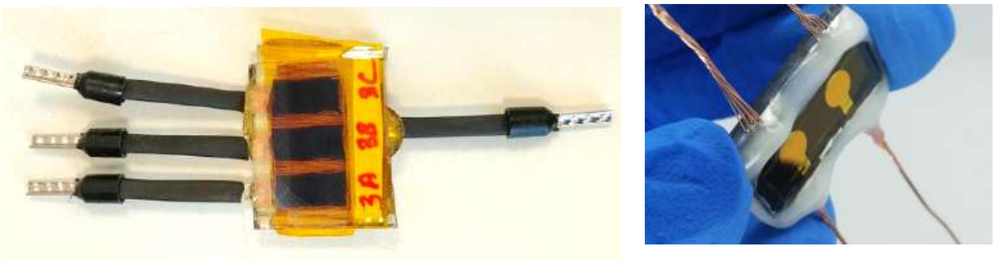

Figure S1. Devices fabricated at BGU (left) and at ICN2 (right).

4. **Indoor and outdoor photostability studies**

The cells were subjected to indoor and outdoor photostability tests at maximum power point (MPP) conditions using a MPP tracker (MP0205M6, University of Ljubljana, Faculty of

Electrical Engineering, Laboratory of Photovoltaics and Optoelectronics). For every test (indoor and outdoor) 4 identical devices were fabricated by ICN2 per annealing temperature and 6 different annealing temperatures were tested which were used to train the model in a cross-validation method. Subsequently, four identical devices were tested outdoor in BGU. The curves were averaged, and the result was compared to that predicted using the environmental conditions and the average of the seven indoor measurements as inputs. In total, the data from stability testing of more than 100 PSC devices from two laboratories was used in this study. As will be detailed below, samples measured indoors were not encapsulated but placed on a cell holder under selected atmosphere (Air or N_2), while samples for the outdoor analyses were always encapsulated.

Indoor Stability under ISOS-L Protocol:

At BGU: Encapsulated cells were mounted on a temperature-controlled (by water circulation) metal plate set at 50 ºC or 15 ºC. The cells were connected to the MPP tracker and the current and voltage data was collected at intervals of 300 s. During this experiment, the cells were illuminated continuously by a solar simulator (SolarConstant MHG 4000/2500 solar simulator, K.H. Steuernagel Lichttechnik GmbH) with an intensity of 100 mW/cm2.

At ICN2: Unencapsulated devices were set under constant illumination (calibrated LED lamp, no UV) and continuous N_2 gas or air flow at maximum power point tracking (MPPT). Stability measurements were carried out at different light intensities.

Outdoor Stability under ISOS-O Protocol:

At BGU: The encapsulated cells were connected to the MPPT and mounted on a dual-axis solar tracker, and the current and voltage data was collected at 30-300 s intervals. The incident solar irradiation and temperature were continuously recorded. The location of the test site was Sede Boqer, Israel (30.8523° N, 34.7834° E) and the dates were 23.02.2023 – 26.03.2023.

At ICN2: Outdoor MPP tracking was performed in Barcelona, Spain (41.3°N, 2.1°W). Encpasulated PSCs covered by a commercia UV filter adhesive film, were mounted on dual-axis trackers, solar intensity was measured with ISO 9060 / IEC 61724 Class A pyranometers while temperature was detected using RK330-01 Atmospheric Temperature, Humidity & Pressure Sensors. All sensors were mounted on the same sun tracker as the PSCs.

Algorithms and Data Processing Methods

1. Algorithm Testing Strategy

To ensure that the limited dataset did not lead to a non representative method evaluation through accidentally beneficial training-test split, we employed a 6-fold validation strategy that is outlined in Figure S2.

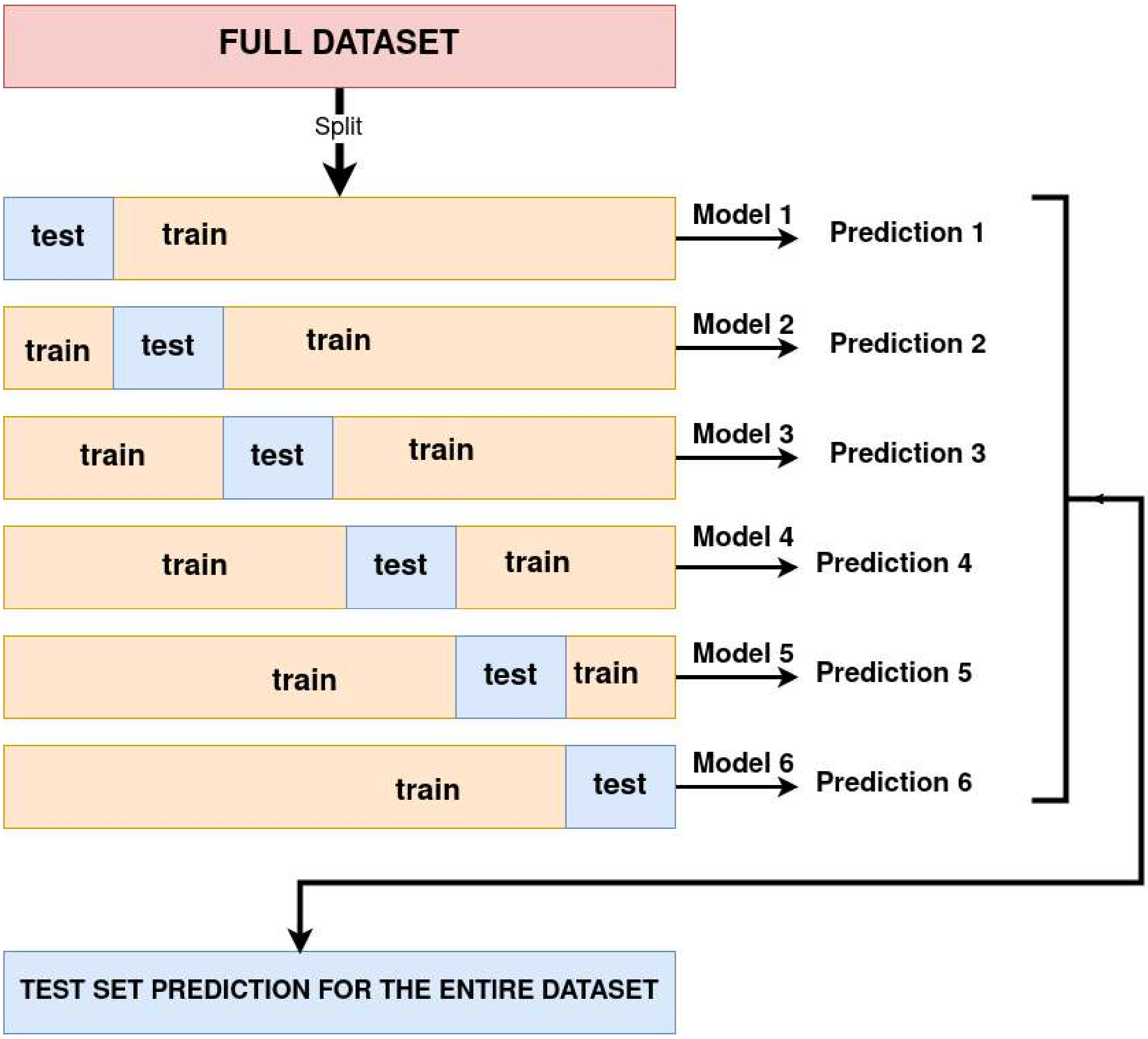

Figure S2. Model Testing Strategy

2. Results of Different Algorithms

The scope and extend of a letter forces the presentation of the best method. Nevertheless, a wide variety of methods has been tests. Gaussian processes for example have been employed and though qualitatively they agree that air is the last relevant as can be seen in Figure S3, their prediction error is significantly higher than that of Kernel Ridge Regression. Nevertheless, the main findings for the relevant indoor tests hold as can be seen in Figure S4. Specifically, that illumination is dominant and air is the least relevant test.

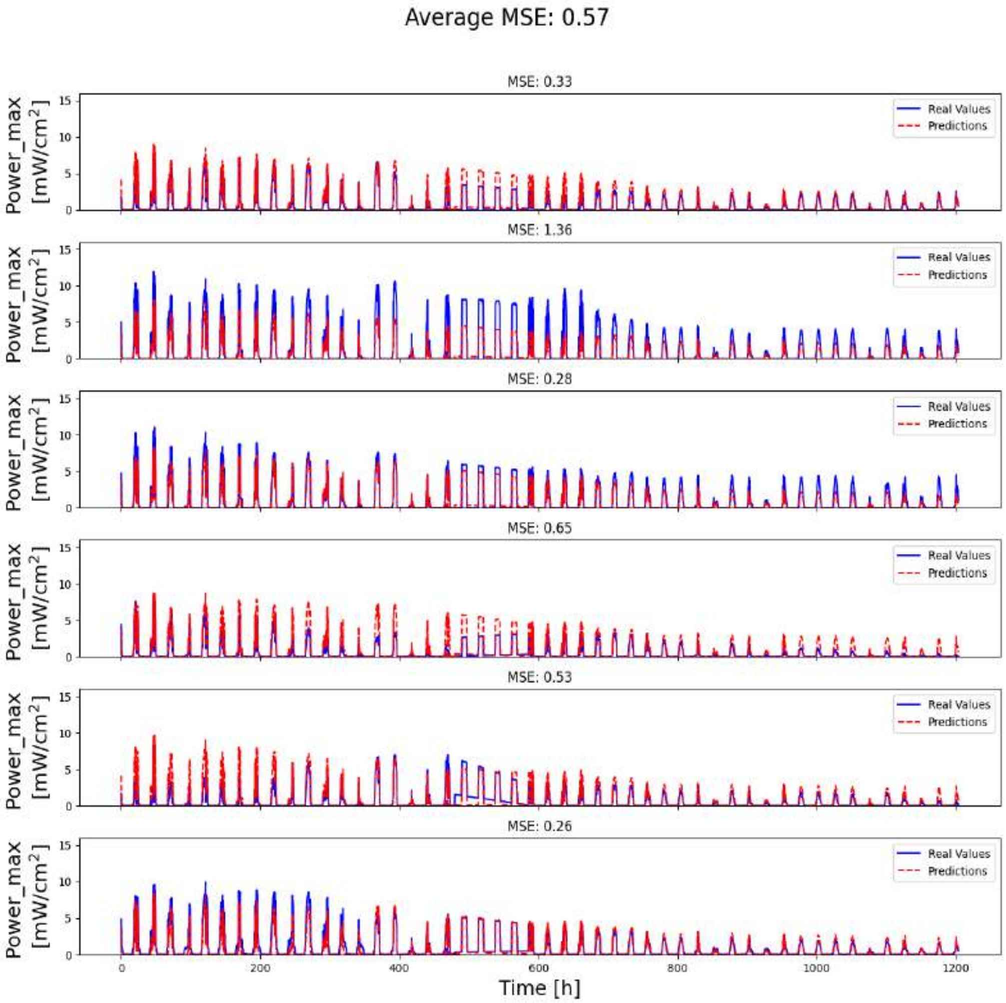

Figure S3. Prediction of Outdoor behaviour using Gaussian Processes

Finally, two additional algorithms were tried, namely bidirectional Long Short Term Memory Networks and Transformers.

Even though their potential is much higher, the limited number of data points at present prevent them from being properly trained. It is therefore not surprising that their predictions are essentially recreating the time series mean, a telltale sign of a model that hasn't learned, as can be seen in Figure S5. Nevertheless, laboratories with high throughput capacities would strongly benefit from these algorithms as they are expected to strongly outperform the relatively simple predictors we used, given enough training data.

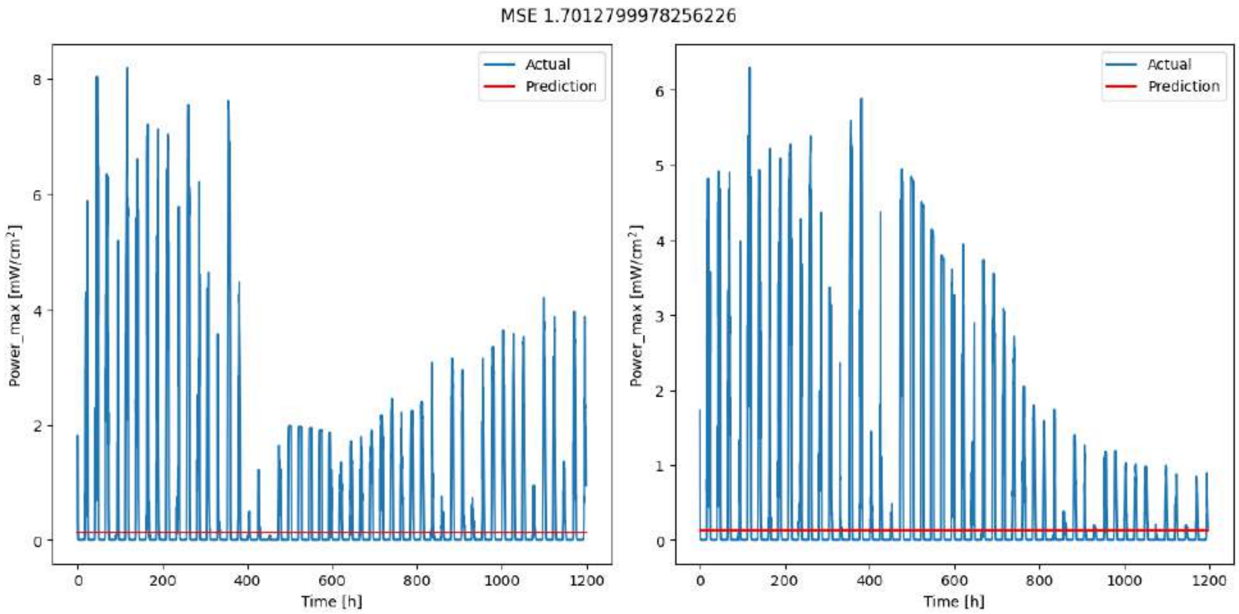

Figure S5. Prediction of outdoor behaviour based on Transformers

References

[1]Michael Saliba, Juan-Pablo Correa-Baena, Christian M. Wolff, Martin Stolterfoht, Nga Phung, Steve Albrecht, Dieter Neher, and Antonio Abate, *How to Make over 20% Efficient Perovskite Solar Cells in Regular (n–i–p) and Inverted (p–i–n) Architectures,* Chem. Mater. 2018, 30, 13, 4193–4201
[2]Karimipour, M.; Paingott Parambil, A.; Tabah Tanko, K.; Zhang, T.; Gao, F.; Lira-Cantu, M. Functionalized MXene/Halide Perovskite Heterojunctions for Perovskite Solar Cells Stable Under Real Outdoor Conditions. *Advanced Energy Materials* 2023, *13* (44) 230195
